# Heart Rate Variability: A Novel Modality for Diagnosing Neuropathic Pain after Spinal Cord Injury

**DOI:** 10.3389/fphys.2017.00495

**Published:** 2017-07-18

**Authors:** Jay Karri, Larry Zhang, Shengai Li, Yen-Ting Chen, Argyrios Stampas, Sheng Li

**Affiliations:** ^1^Department of Physical Medicine and Rehabilitation, The University of Texas Health Science Center at Houston Houston, TX, United States; ^2^TIRR Memorial Hermann Research Center, TIRR Memorial Hermann Hospital Houston, TX, United States

**Keywords:** heart rate variability, neuropathic pain, spinal cord injury, autonomic dysfunction, human

## Abstract

**Background:** Heart rate variability (HRV), the physiological variance in the heart's R-R interval length, can be analyzed to produce various parameters reflective of one's autonomic balance. HRV analysis may be used to capture those autonomic aberrations associated with chronic neuropathic pain (NP) in spinal cord injury (SCI). This study assesses the capacity of HRV parameters to diagnose NP in an SCI cohort.

**Methods:** An electrocardiogram (ECG) was collected at rest from able bodied participants (AB, *n* = 15), participants with SCI only (SCI-NP, *n* = 11), and those with SCI and NP (SCI+NP, *n* = 20). HRV parameters were analyzed using conventional time and frequency analysis.

**Results:** At rest, there were no heart rate differences amongst groups. However, SCI+NP participants demonstrated lower overall HRV, as determined by the SDNN time domain parameter, compared to either AB (*p* < 0.01) or SCI-NP (*p* < 0.05) groups. Moreover, AB and SCI-NP participants were statistically comparable for all HRV time and frequency domain parameters. Additional analyses demonstrated no differences in HRV parameters between T4, above vs. T5, below SCI groups (for all parameters: *p* > 0.15) or between C8, above vs. T1, below SCI groups (*p* > 0.30).

**Conclusions:** Participants with SCI and NP exhibit a lower overall HRV, which can be determined by HRV time domain parameter SDNN. HRV analysis is an innovative modality with the capacity for objective quantification of chronic NP in participants with SCI.

## Background

Spinal cord injury (SCI) is a devastating condition that affects thousands of persons in the United States every year (Siddall et al., 2003). In addition to significant motor impairments, persons with SCI are susceptible to a plethora of systemic complications ranging from bowel and bladder dysfunction to chronic pain conditions (Jensen et al., 2007a,b). Chronic pain conditions in this population are commonly reported to negatively impact mood, functional independence, and quality of life (Jensen et al., 2007b). Unfortunately, managing pain in persons with SCI is clinically challenging, in part due to existing numerous presentations, often concomitantly (O'Connor and Dworkin, 2009; Bryce et al., 2012). The International Spinal Cord Injury Pain classification categorizes pain etiologies as being “nociceptive” for largely musculoskeletal or visceral symptomology, “neuropathic” for spinal cord or nerve derived symptomology, “other” for less common symptomology including fibromyalgia and complex regional pain syndrome, or “unknown” (Bryce et al., 2012). Although above chronic pain conditions contribute greatly to patient suffering, neuropathic pain after SCI has been reported to be especially distressing (Jensen et al., 2007b; O'Connor and Dworkin, 2009; Bryce et al., 2012). Prevalent in up to 85% of patients with SCI, neuropathic pain (NP) is a chronic pain presentation that is challenging to treat, partially related to the fact that mechanisms of NP pathophysiology are poorly understood (Urch, 2011; Margolis et al., 2014). Moreover, many current pharmacological interventions are associated with numerous adverse outcomes including addiction, withdrawal, sedation, and constipation (O'Connor and Dworkin, 2009). Additionally, many patients even report their pain to persist or worsen over time even with pharmacological intervention (O'Connor and Dworkin, 2009; Urch, 2011; Margolis et al., 2014).

Our prevailing understanding of pain physiology is derived from the multi-dimensional pain theory, which suggests the presence of somatic, affective, and evaluative components of pain (Urch, 2011; Garland, 2012). The somatic component is resultant of immunochemical mediators produced by the actual tissue trauma. The affective component corresponds to the emotional unpleasantness of pain necessary to recruit the autonomic nervous system and produce a homeostatic response (Appelhans and Luecken, 2008; Urch, 2011; Garland, 2012). The evaluative component involves the cognitive appraisal of pain stimuli, which is associated with a physiological elevation of sympathetic tone (Garland, 2012). Chronic NP is thought to largely involve the affective and evaluative components and be highly associated with aberrations in the autonomic nervous system (Appelhans and Luecken, 2008; Urch, 2011; Garland, 2012). It has been shown that pain-induced activation of cortical areas, such as the insular cortex and anterior cingulate cortex (ACC) are part of the central autonomic network (CAN) (Hui et al., 2000). The CAN also includes the amygdala, hypothalamus, periaqueductal gray matter, parabrachial complex, the nucleus tractus solitaries, and ventrolateral medulla (Benarroch, 1993; Napadow et al., 2008; Beissner et al., 2013). Given the shared areas involved in the central autonomic network and pain processing network, correlations between pain experience and autonomic responses are expected and confirmed in a recent study by Seifert et al. (2013).

SCI leads to interruptions of ascending and descending pathways to and from the brain as well as inputs to the spinal cord from the periphery, i.e., partial/completed denervation of spinal neurons. Neuroplasticity at the injury level can lead to recovery, but may also contribute to the development of NP. Maladaptive neuroplasticity and subsequent negative outcomes, such as collateral sprouting, astrocytic/microglia activation, and loss of descending inhibition, may lead to sensitization of the central nervous system and subsequent hyperexcitability and central pain (Brown and Weaver, 2012; Finnerup, 2013; Watson and Sandroni, 2016). This view is supported by the findings in a recent longitudinal study in which the authors reported that sensory hypersensitivity (mechanical allodynia and temporal summation of pain) preceded development of central pain below the injury level in SCI participants with incomplete injury (Zeilig et al., 2012). In chronic complete thoracic SCI participants with persistent below-level NP, a normal cognitive task of imagined foot movements evoked an increase in pain or even induced pain in an area that was not previously painful (Gustin et al., 2010). In addition to activity in imagery-related cortical areas as observed in control subjects, SCI participants increased activity in the shared central neural networks between the pain processing network and central autonomic network, including the insular cortex and ACC (Gustin et al., 2010). Chronic NP is thought to largely involve the affective and evaluative components and be highly associated with aberrations in the autonomic nervous system (Appelhans and Luecken, 2008; Bennett, 2010).

The notion of a shared central network between pain processing and autonomic system provides a theoretical basis to make objective diagnosis of chronic NP possible. The current standard for pain quantification, the visual analog scoring system, is highly subjective and largely ineffective (Boonstra et al., 2008). Heart rate variability (HRV), the physiological variance in the heart's inter-beat interval (R-R length), correlates with autonomic balance, thus a potential candidate as a biomarker for diagnosis of chronic NP (Sztajzel, 2004). HRV analysis via a time domain approach provides numerous values including the standard deviation in R-R length (SDNN), root mean squared of successive differences (RMSSD), pairs of successive R-R beat lengths that differ by more than 50 ms (NN50), and the proportion of NN50 for total number of beats (pNN50) (1996). While SDNN is a measure of overall HRV and is sensitive to both sympathetic and parasympathetic modulations, the remainder of HRV time domain parameters have been evidenced to reflect the degree of parasympathetic tone (1996; Koenig et al., 2014). Lower overall HRV, as determined by lower values of SDNN, is thought to be reflective of a decrease in parasympathetic activity and/or an increase in sympathetic activity. Additionally, a frequency domain analysis of HRV dichotomizes inter-beat interval frequencies into low frequency (LF) or high frequency (HF) bands, both of which also reflect the degree of parasympathetic tone (1996; Reyes del Paso et al., 2013; Koenig et al., 2014).

Prior research in HRV has found patients with acute and chronic somatic pain syndromes to exhibit an elevated sympathetic tone (Storella et al., 1999; Koenig et al., 2014). The use of HRV to diagnose NP, however, has had little attention. This is even more intriguing whether HRV parameters could be used as biomarkers of NP after SCI, since recent studies demonstrated autonomic dysfunction and abnormal HRV in pain-free persons with SCI (Malmqvist et al., 2015; Serra-Añó et al., 2015; Rodrigues et al., 2016). Therefore, the primary aim of our study was to determine if baseline HRV parameters in a resting state could be used to diagnose NP in an SCI cohort. Moreover, we also aimed to explore if other relevant factors like demographics, baseline heart rhythm variables, or SCI clinical parameters were responsible for potential autonomic aberrations. Essentially, we hope to elucidate if HRV differences were produced by NP status alone or subject to confounding by the aforementioned factors that may carry significant cardiac influences.

Similar to previous findings of decreased HRV and lower parasympathetic tone in other pain conditions, we hypothesized that participants with SCI and NP demonstrate a low parasympathetic tone irrespective of clinically relevant SCI parameters including time since injury, completeness of injury, or level of SCI (Adeyemi et al., 1999; Storella et al., 1999; Cohen et al., 2000; Kalezic et al., 2007). We further hypothesized that HRV parameters could capture the aforementioned decreased parasympathetic tone and diagnose baseline NP in an SCI cohort.

## Methods

### Participant population

This study was carried out in accordance with the recommendations of the institutional review board at the University of Texas Health Sciences Center at Houston with written informed consent received from all participants. Both SCI and able-bodied participants were recruited in this study. Criteria for able-bodied participants (AB) were persons (1) between 18 and 75 years of age, (2) capable of providing consent, (3) without clinically significant or unstable medical, neuropsychiatric (depression in particular), or chronic pain disorders, (4) without a history of substance abuse or dependence, (5) without a history of brain surgery, intracranial metal implantation, or tumor, and (6) without a history of cardiac pathology, implanted pacemakers, or current use of rhythm altering medication like beta-blockers. In addition to those criteria used for AB, participants with SCI were recruited into either the SCI without NP study group (SCI-NP) or SCI with NP study group (SCI+NP) if they were diagnosed with an SCI at least 6 months prior. SCI-NP participants were further required to be pain free or have a nociceptive pain condition, i.e., a pain disorder other not deemed NP, often musculoskeletal in origin. SCI+NP participants were required to (1) have a SCI for at least 6 months, (2) have chronic NP for >3 months, as diagnosed by a SCI medicine board certified physician, (3) have stable pain symptoms and analgesic medications for at least 2 weeks prior to the experiment. Participants were excluded from the study if they (1) were currently adjusting oral pain medications, or (2) were suspected to have autonomic dysreflexia in the 24 h preceding testing. Recruitment was based from our specialty clinic by convenience.

### Experimental details

To limit circadian influences, all HRV data collection occurred in the early afternoon between 1PM and 3PM. Participants were escorted to a research laboratory, and monitored in a seated position for 5 min to ensure their being in a comfortable and resting state prior to beginning the study. The participant's skin was then cleaned with an alcohol wipe to ensure appropriate electrode contact. After attaching disposable adhesive electrodes to each ECG lead, the white “right arm” and black “left arm” electrode leads were placed along the 1st intercostal spaces in the subclavicular area on the right and left chest wall, respectively. The red “left leg” electrode was placed along the lower intercostal spaces on the left mid-axillary line. After instructions to remain seated, calm, and relaxed with limited movement, a 5-min ECG recording was collected at resting baseline for all participants using a heart rhythm scanner (Biocom 5000 Wireless ECG Recorder, Biocom Technologies, Poulsbo, Washington). ECG signals were saved for off-line HRV analysis.

### Data analysis

All demographic data were provided by the participants. The electronic medical record was surveyed for the most recent International Standards for Neurological Classification of SCI exam documented by an SCI specialized physician to gather SCI associated clinical variables, which include the time since injury, neurologic injury level, and severity of injury. A medication reconciliation of active prescription pain medications in the electronic medical record determined the type of medications being used, at what frequency and dosages, and for which indications. Pain medications were categorized as GABA analogs, which include gabapentin and pregabalin, atypical antidepressants, which commonly include tricyclic antidepressants (TCA) and serotonin-norepinephrine reuptake inhibitiors (SNRI), and opiates, for which morphine equivalents were reported as is conventional (Gustin et al., 2010).

Kubios HRV analysis software (University of Eastern Finland, Joensuu, Finland) was used to evaluate the ECG recording via time and frequency domain approaches to obtain various HRV parameters. The time domain HRV parameters included SDNN, RMSSD, NN50, and pNN50. The frequency domain parameters included LF, HF, and LF/HF; these parameters were collected based on the standard frequency stratification designating LF as 0.04–0.15 Hz and HF as 0.15–0.40 Hz, as determined by the fast Fourier transformation algorithm.

### Statistical analysis

The major dependent variables for demographics were (1) age and (2) gender; for participants with SCI, the major dependent variables were (1) years since injury, (2) prevalence of complete injury and (3) presence of a neurologic injury level of T4 and above or C8 and above. The major HRV dependent variables for time domain analysis were (1) SDNN, (2) RMSSD, (3) NN50, and (4) pNN50; the major HRV dependent variables for frequency domain analysis were LF, HF, and LF/HF. We chose to analyze groups above and below the neurologic level of T4 to evaluate the role of intact sympathetic nerve supply to the heart. An additional analysis was conducted comparing groups above and below the neurological level of C8 to assess for the role of tetraplegia (C8 and above) and paraplegia (T1 and below) on potential autonomic aberrations.

Non-parametric Kruskal-Wallis and Mann-Whitney *U*-tests were used to determine among and between group differences, respectively, in non-Gaussian distributed parameters (SDNN, RMSSD, pNN50, LF, HF, and LF/HF). Significant differences amongst groups, determined by Kruskal-Wallis tests, were further interrogated with non-parametric ranksum pairwise testing with Bonferroni corrections. For all other parameters, one-way ANOVA analysis and independent samples *t*-test analysis were used to determine differences in demographics, SCI relevant clinical parameters, and NN50 when comparing three and two study groups, respectively. Statistical analysis was performed using the STATA Version 12.1 (StataCorp LP, College Station, TX). An alpha level of 0.05 was used as threshold for significance for all statistical tests. Data were reported as mean ± SD within the text and as mean ± SEM in the figures. Only the significant main effects were presented, unless otherwise noted.

## Results

### Demographics

Our study cohort included participants stratified into AB (*n* = 15), SCI-NP (*n* = 11), or SCI+NP (*n* = 20) study groups. Overall, all three groups exhibited similar demographics including age and gender (Table 1). Both SCI-NP and SCI+NP groups showed similar clinical parameters, including years since injury, incidence of C8 and above, T4 and above the neurologic level of injury, and complete injury prevalence. In regards to neuropathic pain treatment, only three participants in the SCI-NP group utilized pain medication, and all for indications other than NP; no medications besides gabapentin were utilized (Table 2). In the contrary, the majority of participants in the SCI+NP group utilized medications, and all for indications of NP (Table 3). Additionally, they utilized not only higher dosages of gabapentin, but also other classes of medications including atypical antidepressants and opiates. TCA medications utilized included amitriptyline (participant 4, 19) and nortriptyline (participant 15) and SNRI medications utilized included venlafaxine (participant 2) and duloxetine (participant 20). Of note, participant 3 was not utilizing any pain medications secondary to poor tolerance to GABA analogs.

**Table 1:** Displayed are the values for pertinent demographic, SCI-relevant, heart rhythm, time domain, and frequency domain parameters for all three participants groups (AB: able bodied participants, SCI-NP: participants with SCI only, SCI+NP: participants with SCI and chronic NP).

	**Able-bodied (AB) (*N* = 15)**	**SCI–NP (*N* = 11)**	**SCI + NP (*N* = 20)**	***p*-value**
**DEMOGRAPHICS**				
Age (years)	37.73 ± 16.46	38.55 ± 15.55	44.89 ± 14.05	*P* = 0.471, F: 0.77
Male (%)	53.33 ± 51.64	72.72 ± 46.71	88.89 ± 32.34	*P* = 0.125, F: 2.19
**SCI PARAMETERS**				
Years since injury	–	11.41 ± 11.67	9.92 ± 10.13	*P* = 0.633, T: 0.34
C8 and above (%)	–	36.37 ± 50.45	66.67 ± 48.51	*P* = 0.134, T: –1.54
T4 and above (%)	–	54.55 ± 52.22	66.67 ± 48.51	*P* = 0.583, T: –0.56
Complete injury (%)	–	27.27 ± 46.71	38.89 ± 50.16	*P* = 0.349, T: –0.95
**BASELINE HEART RHYTHM**				
Average heart rate (beats per min.)	70.41 ± 9.91	63.59 ± 11.79	72.97 ± 12.87	*P* = 0.118, F: 2.25
Mean R-R length (milliseconds)	871.70 ± 110.97	982.51 ± 208.89	849.26 ± 148.37	*P* = 0.076, F: 2.74
**TIME DOMAIN PARAMETERS**				
SDNN (milliseconds)	62.22 ± 20.59	60.15 ± 33.65	37.3 ± 23.88	*P* = 0.003, ^2^: 11.45
RMSSD (milliseconds)	52.04 ± 21.18	43.43 ± 36.71	20.06 ± 9.43	*P* ≤ 0.001, *X*^2^: 22.13
NN50 (count)	83.53 ± 54.19	38.73 ± 50.05	16.78 ± 28.50	*P* ≤ 0.001, F: 10.53
pNN50 (%)	24.53 ± 16.85	13.51 ± 17.31	4.79 ± 7.49	*P* ≤ 0.001, *X*^2^: 16.13
**FREQUENCY DOMAIN PARAMETERS**				
LF (power ms^2^)	945.00 ± 647.71	783.91 ± 1308.08	379.89 ± 530.15	*P* = 0.016, *X*^2^: 8.22
HF (power ms^2^)	1104.33 ± 998.23	714.27 ± 1168.01	232.61 ± 335.49	*P* = 0.001, *X*^2^: 13.23
LF/HF	1.20 ± 1.01	1.37 ± 0.67	2.60 ± 3.30	*P* = 0.310, *X*^2^:2.34

*Time domain variables include SDNN: standard deviation in N-N intervals, RMSSD: root mean squared of successive differences, NN50: pairs of successive R-R beat lengths varying by greater than 50 ms, pNN50: proportion of NN50 for total number of pNN50. SDNN reflects overall HRV, while RMSSD, NN50, and pNN50 reflect parasympathetic tone. Frequency domain variables include LF: low frequency band, HF: high frequency band, and LF/HF: low to high frequency ratio. LF and HF reflect parasympathetic tone, while LF/HF reflects degree of autonomic balance. P-values were determined by one way ANOVA and independent samples t-tests for Gaussian distributed parameters (demographics, SCI parameters, baseline heart rhythm, SDNN) compared amongst 3 groups and between 2 groups, respectively. For all non-Gaussian distributed parameters (time and frequency domain parameters except SDNN), Kruskal Wallis and Mann-Whitney U-tests were used to compare 3 groups and 2 groups, respectively*.

**Table 2:** Displayed are the values for pertinent demographics, SCI relevant parameters, and pain medications, along with their dosage, frequency, and indications, for each participant in the SCI-NP cohort (participants with SCI only).

**Participant**	**Age**	**Gender**	**Years since injury**	**Injury level, Asia score**	**Pain medications (mg)**	**Medication indication**
1	37	M	17	C4, B	N/A	N/A
2	46	M	1.5	C5, C	N/A	N/A
3	19	F	1	C7, C	N/A	N/A
4	35	M	16.5	C8, A	N/A	N/A
5	21	F	2.5	T2, A	Gabapentin 300q8h	PRN, for menses
6	22	M	4.5	T4, C	N/A	N/A
7	61	M	40	T8, B	N/A	N/A
8	64	M	20	T9, D	N/A	N/A
9	48	M	8.5	T12, A	N/A	N/A
10	25	M	3	T11, C	Gabapentin 300q8h	PRN, for constipation
11	46	F	11	T12, C	Gabapentin 100q12h	PRN, for menses

**Table 3:** Displayed are the values for pertinent demographics, SCI relevant parameters, and pain medications, along with their dosage and frequency, for each participant in the SCI+NP cohort (participants with SCI and chronic NP).

**Participant**	**Age**	**Gender**	**Years since injury**	**Injury level, Asia score**	**Gaba analogs (mg)**	**Atypical antidepressants (mg)**	**Daily morphine equivalents (mg)**	**Pain location**	**VAS score**
1	36	M	1	C1, B	N/A	N/A	30	Legs	5.0
2	28	M	1	C4, A	Gabapentin 300q24h	Venlafaxine 37.5q12	N/A	Buttocks, thighs	4.0
3	55	M	9	C4, C	N/A	N/A	N/A	Thighs to feet	3.5
4	32	M	10	C5, A	Gabapentin 400q8h	Amitriptyline 25q24h	40	Hips, buttocks	5.0
5	54	M	37	C5, C	Gabapentin 300q8h	N/A	N/A	Calves, feet	6.0
6	47	M	11	C5, C	Gabapentin 600q8h	N/A	N/A	Hips, thighs	4.0
7	59	M	8	C5, D	Gabapentin 600q8h	N/A	N/A	Hips to feet	4.0
8	31	M	5	C6, A	Gabapentin 300q8h	N/A	N/A	Hips to feet	3.0
9	24	M	8	C6, B	N/A	N/A	20	Shoulders, lumbar back	3.0
10	37	M	12	C6, A	Gabapentin 300q8h	N/A	N/A	Hips to thighs	3.5
11	61	M	14	C6, A	Gabapentin 500q8h	N/A	N/A	Thoracic, lumbar back	4.0
12	66	F	25	C6, C	Gabapentin 300q12h	N/A	N/A	Lumbar back, hips to feet	7.5
13	50	M	27	C6, D	N/A	N/A	PRN, 5-10	Hips to feet	7.0
14	28	F	11	T7, A	Gabapentin 300q8h	N/A	N/A	Lumbar back, flank	5.0
15	30	M	1	T10, A	Gabapentin 800q6h	Nortriptyline 25q12h	N/A	Thighs, feet	4.5
16	26	M	1	T10, A	Gabapentin 600q8h	N/A	N/A	Lumbar back, hips	5.0
17	40	M	2.5	T12, D	Gabapentin 600q8h	N/A	N/A	Calves, feet	5.0
18	61	M	4	L1, A	Pregabalin 50q12h	N/A	N/A	Thighs, feet	6.5
19	59	F	4	L2, D	N/A	Amitriptyline 50q24h	20	Waistband, hips to feet	7.0
20	49	M	10	L2, D	Pregabalin 150q8h	Duloxetine 60q24h	N/A	Buttocks, thighs, feet	3.5

*Note that opoid medications were converted to morphine equivalents*.

### HRV differences

**Figure 1:**
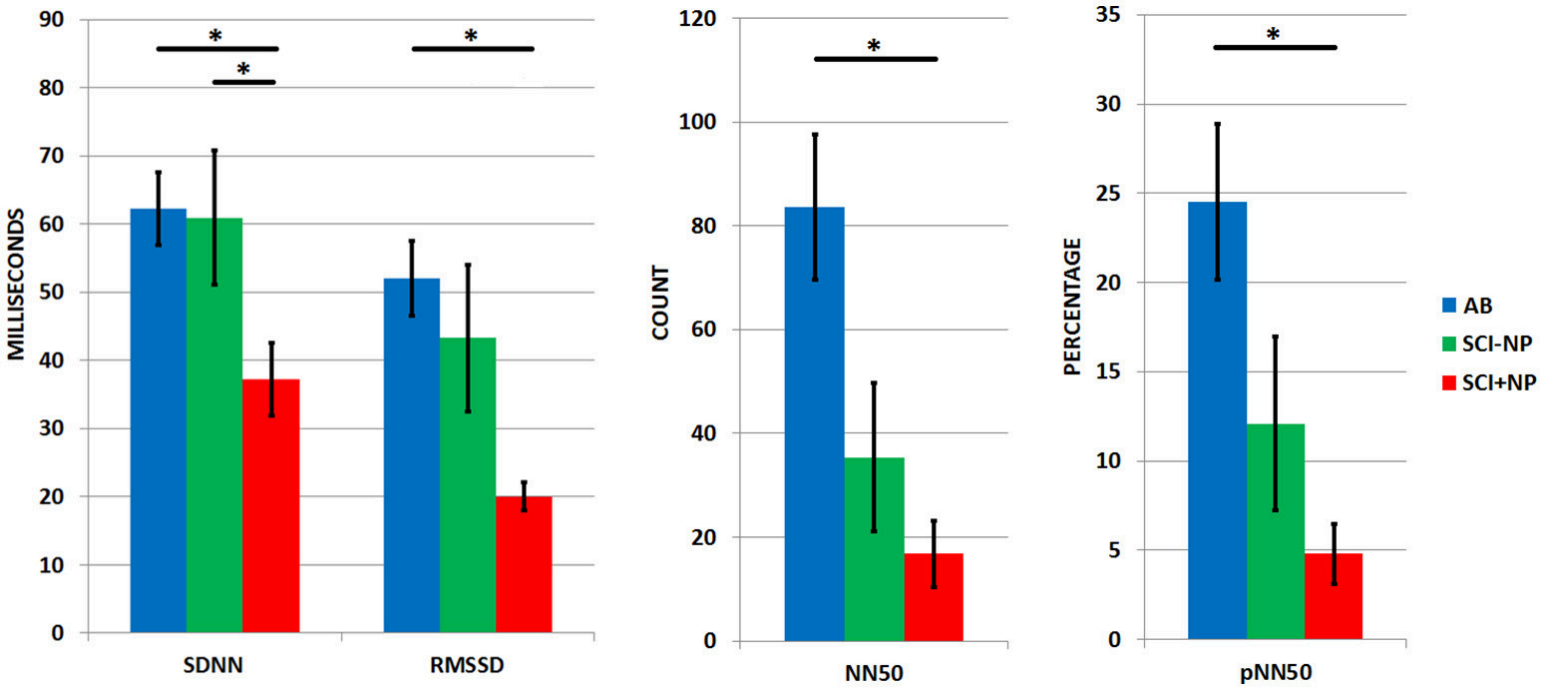
HRV time domain parameters for all three groups (AB: able bodied participants, SCI-NP: participants with SCI only, SCI+NP: participants with SCI and chronic NP) with means and SEM shown. Asterisks denote statistically significant differences. Parameters displayed include SDNN: standard deviation in N-N intervals, RMSSD: root mean squared of successive differences, NN50: pairs of successive R-R beat lengths varying by greater than 50 ms, pNN50: proportion of NN50 for total number of pNN50. SDNN reflects overall HRV, while RMSSD, NN50, and pNN50 reflect parasympathetic tone.

The resting average heart rate and average R-R interval length were similar amongst the three groups (Table 1). On the other hand, differences were appreciated amongst the three groups for all time domain parameters (Table 1 and Figure 1). Namely, pairwise Mann-Whitney *U*-test comparisons with Bonferroni corrections determined SCI+NP participant to exhibit a significantly lower overall HRV as evidenced by lower values for the SDNN time domain parameter compared to corresponding values in AB (*p* < 0.01) and SCI-NP groups (*p* < 0.05). There were no differences in SDNN between AB and SCI-NP participants. In regards to the RMSSD, NN50, and pNN50 parameters, the AB groups exhibited a significantly higher parasympathetic tone than the SCI+NP group (*p* < 0.01 for all parameters). There were no differences in RMSSD, NN50, or pNN50 between AB and SCI-NP participants.

**Figure 2:**
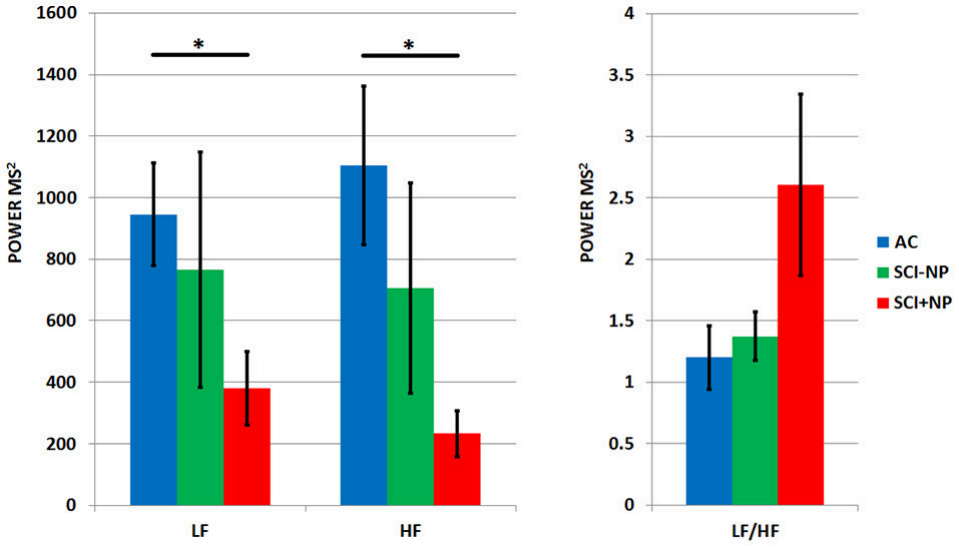
HRV frequency domain parameters for all three groups (AB: able bodied participants, SCI-NP: participants with SCI only, SCI+NP: participants with SCI and chronic NP) with means and SEM shown. Asterisks denote statistically significant differences. Parameters displayed include LF: low frequency band, HF: high frequency band, and LF/HF: low to high frequency ratio. LF and HF reflect parasympathetic tone, while LF/HF reflects degree of autonomic balance.

For the frequency domain parameters, differences amongst the three groups were found for the LF and HF parameters (Table 1 and Figure 2). Pairwise Mann-Whitney *U*-test comparisons with Bonferroni corrections determined that the AB group exhibited a higher parasympathetic tone, in regards to LF and HF parameters, than the SCI+NP group (*p* < 0.02 for both parameters). There were no differences between either AB vs. SCI-NP or SCI+NP vs. SCI-NP group comparisons for all frequency domain parameters.

To assess for the potential role of intact spinal derived sympathetic innervation to the heart, all SCI participants were also compared in groups of T4 and higher (*n* = 19) vs. T5 and lower (*n* = 12), irrespective of the presence of NP (Table 4). Reanalysis for these new groups did not demonstrate differences in demographics, SCI relevant parameters, or baseline heart rhythms. Moreover, there were no differences in regards to any time or frequency domain HRV parameters. As evidenced by Table 4, the statistical homogeneity between the two groups can be appreciated amongst all variables.

**Table 4:** Displayed are the values for pertinent demographic, SCI-relevant, heart rhythm, time domain, and frequency domain parameters for the T4, above and T5, below participant groups.

	**T4, above (*N* = 19)**	**T5, below (*N* = 12)**	***p*-value**
**DEMOGRAPHICS**			
Age (years)	40.00 ± 14.56	44.75 ± 14.75	0.394
Male (%)	84.21 ± 37.46	75.00 ± 45.23	0.685
**SCI PARAMETERS**			
Years since injury	11.11 ± 9.91	9.67 ± 11.05	0.584
Complete injury (%)	36.84 ± 49.56	41.67 ± 51.49	0.839
**BASELINE HEART RHYTHM**			
Average heart rate (beats per min.)	68.62 ± 14.17	70.91 ± 11.11	0.584
Mean R-R length (milliseconds)	916.64 ± 199.60	867.95 ± 144.93	0.556
**TIME DOMAIN PARAMETERS**			
SDNN (milliseconds)	53.23 ± 34.61	34.94 ± 10.95	0.162
RMSSD (milliseconds)	31.94 ± 30.79	21.97 ± 10.62	0.453
NN50 (count)	29.42 ± 43.48	15.83 ± 25.61	0.641
pNN50 (%)	9.36 ± 13.96	5.35 ± 9.00	0.715
**FREQUENCY DOMAIN PARAMETERS**			
LF (power ms^2^)	669.74 ± 1087.22	348.75 ± 315.32	0.984
HF (power ms^2^)	696.05 ± 1200.71	227.92 ± 274.81	0.556
LF/HF	2.14 ± 3.27	2.19 ± 1.32	0.128

*Time domain variables include SDNN: standard deviation in N-N intervals, RMSSD: root mean squared of successive differences, NN50: pairs of successive R-R beat lengths varying by greater than 50 ms, pNN50: proportion of NN50 for total number of pNN50. SDNN reflects overall HRV, while RMSSD, NN50, and pNN50 reflect parasympathetic tone. Frequency domain variables include LF: low frequency band, HF: high frequency band, and LF/HF: low to high frequency ratio. LF and HF reflect parasympathetic tone, while LF/HF reflects degree of autonomic balance. P-values were determined by independent samples t-tests for Gaussian distributed parameters (demographics, SCI parameters, baseline heart rhythm, SDNN) and by Mann-Whitney U-tests for non-Gaussian distributed parameters (time and frequency domain parameters except SDNN)*.

Similarly, we further compared all participants with SCI in groups of C8, above (*n* = 17) and T1, below (*n* = 14), irrespective of the presence of NP to assess for the role of tetraplegic status on potential autonomic aberrations (Table 5). This reanalysis also failed to demonstrate any notable differences in demographics, SCI relevant parameters, baseline heart rhythms, and HRV time and frequency domain parameters.

**Table 5:** Displayed are the values for pertinent demographic, SCI-relevant, heart rhythm, time domain and frequency domain parameters for the C8, above and T1, below participant groups.

	**C8, above (*N* = 17)**	**T1, below (*N* = 14)**	***p*-value**
**DEMOGRAPHICS**			
Age (years)	42.18 ± 13.80	41.43 ± 15.98	0.843
Male (%)	88.24 ± 33.21	71.42 ± 46.88	0.439
**SCI PARAMETERS**			
Years since injury	12.00 ± 10.11	8.79 ± 10.41	0.302
Complete injury (%)	35.29 ± 49.26	42.86 ± 51.36	0.736
**BASELINE HEART RHYTHM**			
Average heart rate (beats per min.)	68.07 ± 14.93	71.26 ± 10.26	0.416
Mean R-R length (milliseconds)	927.45 ± 208.90	861.78 ± 134.24	0.393
**TIME DOMAIN PARAMETERS**			
SDNN (milliseconds)	50.81 ± 35.34	40.49 ± 18.67	0.592
RMSSD (milliseconds)	28.86 ± 28.73	27.14 ± 21.13	0.984
NN50 (count)	23.82 ± 35.92	24.57 ± 41.21	0.796
pNN50 (%)	7.92 ± 12.62	7.68 ± 12.31	0.751
**FREQUENCY DOMAIN PARAMETERS**			
LF (power ms^2^)	469.59 ± 634.19	637.64 ± 1125.80	0.487
HF (power ms^2^)	521.71 ± 942.45	506.5 ± 1044.40	0.796
LF/HF	2.27 ± 3.45	2.03 ± 1.28	0.331

*Time domain variables include SDNN: standard deviation in N-N intervals, RMSSD: root mean squared of successive differences, NN50: pairs of successive R-R beat lengths varying by greater than 50 ms, pNN50: proportion of NN50 for total number of pNN50. SDNN reflects overall HRV, while RMSSD, NN50, and pNN50 reflect parasympathetic tone. Frequency domain variables include LF: low frequency band, HF: high frequency band, and LF/HF: low to high frequency ratio. LF and HF reflect parasympathetic tone, while LF/HF reflects degree of autonomic balance. P-values were determined by independent samples t-tests for Gaussian distributed parameters (demographics, SCI parameters, baseline heart rhythm, SDNN) and by Mann-Whitney U-tests for non-Gaussian distributed parameters (time and frequency domain parameters except SDNN)*.

## Discussion

In this study, we collected and analyzed 5-min resting ECG signals using a conventional approach in three groups of research participants: able-bodied participants (AB), participants with SCI and chronic neuropathic pain (SCI+NP), and participants with SCI and without neuropathic pain (SCI-NP). All participants had similar ages and genders were balanced among groups. Participants with SCI had similar clinical parameters such as time since injury and severities of injury (level and completeness). All participants had similar baseline heart rates. The novel and unique findings were that SCI+NP participants demonstrated significantly lower levels of overall HRV (SDNN) as compared to SCI-NP participants and AB participants. There was no effect of level of injury (T4 and above vs. T5 and below or C8 and above vs. T1 and below) on the parasympathetic drive. These findings provide evidence that autonomic activity is a potential biomarker of chronic neuropathic pain in participants after SCI. The findings in this study are consistent with previous findings of altered HRV parameters associated with various pain syndromes, including irritable bowel syndrome, chronic lower back pain, and fibromyalgia (Zeilig et al., 2012; Finnerup, 2013; Watson and Sandroni, 2016). These studies have established similar findings of decreased overall HRV and parasympathetic tone associated with pain in a variety of pain pathologies. Our findings have further confirmed and extended the application of HRV parameters as potential biomarkers of neuropathic pain after SCI.

The physiology of the cardiac cycle with the resulting HRV parameters is sensitive to numerous conditions including age, gender, and autonomic influence (Jensen-Urstad et al., 1997). It can be suggested that varying severities and presentations of SCI can also impact descending autonomic transduction to various extents and thus impact baseline HRV (Karlsson, 2006). Those participants with injuries at the T4 level and higher may have interruptions to spinal derived sympathetic outflow to the heart (Grigorean et al., 2009). Likewise, participants with tetraplegia, caused by injuries at the C8 level and higher may demonstrate a higher relative parasympathetic tone due to intact vagal pathways and disrupted control of supraspinal sympathetic outflow (Takahashi et al., 2007). However, our finding of the lowered overall HRV in SCI+NP participants is a reflection of the status of chronic neuropathic pain. This finding is not likely influenced by spinal cord injury and its potential effects on sympathetic outflow in this study. As demonstrated by the comparison in Table 1, the SCI-NP and SCI+NP groups in our study cohort were statistically homogenous in regards to baseline demographics and SCI associated clinical parameters. These findings are additionally supported by the absence of association between SCI injury level and autonomic influence (Tables 4, 5). Furthermore, the statistical similarity between AB and SCI-NP groups for all time and frequency domain parameters limits the suspicion of SCI pathology alone causing a notable influence on HRV. In the present study, we also found SCI+NP participants to have a non-significant trend toward a lower parasympathetic drive, as determined by lower values for all HRV time domain parameters and LF and HF frequency domain parameters (1996) (Table 1 and Figure 1).

The finding of no effect of injury level of HRV parameters in this study is not trivial. In contrast, previous studies have reported that the presence of higher neurological levels of injury has been found to be independent contributors to a reduced sympathetic tone, as measured by HRV (Malmqvist et al., 2015; Serra-Añó et al., 2015; Rodrigues et al., 2016). In this study, SCI participants with and without NP were recruited. It was not specifically mentioned whether SCI participants with NP were tested or not in these cited studies. However, in the study by Rodriguez et al, no pain medications were listed for their SCI participants, suggesting these participants were without NP (Rodrigues et al., 2016). Given our finding of a significant effect of NP on HRV parameters, it is likely that the NP effects mask or confound the effect of injury level of HRV parameters, in addition to possible effects from medications and sample size (see below).

Certain pain medications could pharmacologically alter HRV physiology. It is possible that HRV of participants with SCI+NP may not be truly representative of their baseline resting status. Congruent with the clinical standard for treating NP associated with SCI, a majority of our SCI+NP cohort utilized GABA analog medications, including gabapentin and pregabalin. These medications, which function by disrupting glutamergic transmission, may produce an increase in parasympathetic tone as intact glutamergic transmission is necessary for appropriate spinal sympathetic initiation (Maiorov et al., 1997). Despite the potential GABA analog driven increase in parasympathetic tone, we still demonstrate that participants with SCI+NP possess a lower overall HRV. Therefore, medication associated confounding of HRV alterations is less likely. Less utilized in our cohort, opiates and atypical antidepressants may also alter HRV. Opiates are thought to increase relative vagal tone to the heart and atypical antidepressant medications, namely SNRIs and TCAs, increase systemic sympathetic tone by way of increasing serotonin and norepinephrine concentrations (Carter et al., 2002; Licht et al., 2012; Yekehtaz et al., 2013). Neither GABA analogs nor opiates are known to carry direct cardiac inotropic effects. Future work in this area may explore the roles of these medications on patients with SCI+NP.

## Study limitations

Our study was powered primarily based on sample sizes of convenience as only participants with chronic SCI were recruited in both SCI+NP and SCI-NP groups. Such small sample sizes limited our effect size and our ability to conduct robust statistical matching. We acknowledge there are limitations in this study. But we believe this is the first step in advancing our understanding of HRV changes in SCI+NP. Findings from our work will help future studies in estimating the appropriate power and sample sizes required. Though the severity of SCI was comparable between two groups, completeness of SCI may be an important contributor to HRV, particular for those at T4 or above.

While the effect size of HRV analysis is greater with longer recordings—with 24 h recordings considered to render the highest quality data, our study utilized only a short-term ECG recording of 5 min for HRV analysis (1996). This time interval was chosen after recent HRV studies investigating pain disorders have deemed a 5 min recording to be sufficient and appropriate in garnering notable autonomic differences (Storella et al., 1999; Appelhans and Luecken, 2008; Broucqsault-Dédrie et al., 2016; Telles et al., 2016). Moreover, we also encountered studies that found longitudinal stability of 5-min analyses compared to 24-h recordings (Min et al., 2008; Telles et al., 2016). Seeing that we were investigating HRV differences in a chronic pain condition, these findings substantiate our choice of using a 5 min recording.

## Future studies

A longitudinal study following acute to chronic HRV changes would provide useful information regarding the natural history of HRV changes in SCI. Additionally, robust large sample data may elucidate whether or how well autonomic normalization may be used as end targets for interventions. Studies exploring other pain disorders have previously shown that analgesic effects can be quantified by increases in parasympathetic modulation, as measured by HRV parameters (Storella et al., 1999; Berry et al., 2014; Yeh et al., 2017). Similarly, HRV parameters may also carry the capacity for objective quantification of analgesic response to NP treatment.

## Conclusion

Diagnosing and treating NP in persons with SCI is clinically challenging. HRV represents an objective modality to diagnose chronic NP in participants with SCI. Using time domain HRV analysis, participants with SCI and chronic NP were found to exhibit a lower overall HRV at rest, as determined by the SDNN parameter. Additional comparisons validated that these HRV differences were resultant of the NP status and were not related to SCI pathology itself. These findings suggest HRV parameters may be biomarkers for neuropathic pain.

## Author contributions

JK, LZ, SAL, YC, and SL designed and performed the experiments. JK, LZ, YC, AS, and SL conducted necessary data analysis and interpretation. JK, YC, SAL, AS, and SL wrote the manuscript.

### Conflict of interest statement

The authors declare that the research was conducted in the absence of any commercial or financial relationships that could be construed as a potential conflict of interest.

## Abbreviations

HRV: heart rate variability
NP: neuropathic pain
SCI: spinal cord injury
ECG: electrocardiogram
AB: able-bodied participants
SCI-NP: participants with spinal cord injury and without neuropathic pain
SCI+NP: participant s with spinal cord injury and with neuropathic pain
SDNN: standard deviation in R-R interval length
RMSSD: root mean squared of successive differences
NN50: pairs of successive R-R beat lengths that differ by more than 50 ms
pNN50: the proportion of NN50 for total number of beats
LF: low frequency
HF: high frequency
LF/HF: ratio of low to high frequency
T4: fourth thoracic level of the spinal cord
C8: eight cervical level of the spinal cord (region where nerve roots exit between the C7 and T1 levels)
T12: twelfth thoracic level of the spinal cord
L1: first lumbar level of the spinal cord
SD: standard deviation
SEM: standard error of the mean
TCA: tricyclic antidepressant
SNRI: serotononin-norepinephrine reuptake inhibitor.
